# Dopamine Receptors and Parkinson's Disease

**DOI:** 10.1155/2011/403039

**Published:** 2011-06-13

**Authors:** Shin Hisahara, Shun Shimohama

**Affiliations:** Department of Neurology, Sapporo Medical University, Sapporo 060-8556, Japan

## Abstract

Parkinson's disease (PD) is a progressive extrapyramidal motor disorder. Pathologically, this disease is characterized by the selective dopaminergic (DAergic) neuronal degeneration in the substantia nigra. Correcting the DA deficiency in PD with levodopa (L-dopa) significantly attenuates the motor symptoms; however, its effectiveness often declines, and L-dopa-related adverse effects emerge after long-term treatment. Nowadays, DA receptor agonists are useful medication even regarded as first choice to delay the starting of L-dopa therapy. In advanced stage of PD, they are also used as adjunct therapy together with L-dopa. DA receptor agonists act by stimulation of presynaptic and postsynaptic DA receptors. Despite the usefulness, they could be causative drugs for valvulopathy and nonmotor complication such as DA dysregulation syndrome (DDS). In this paper, physiological characteristics of DA receptor familyare discussed. We also discuss the validity, benefits, and specific adverse effects of pharmaceutical DA receptor agonist.

## 1. Introduction

Parkinson's disease (PD) is a common progressive neurodegenerative disorder that can be accurately diagnosed. A recent meta-analysis study indicated that standardized all-age prevalence of 51.3 to 176.9 per 100 000 in door-to-door surveys and prevalence in record-based studies ranged from 35.8 to 68.3 per 100 000 in Asia [1]. The standardized incidence rates were 8.7 per 100 000 person-years in door-to-door surveys and 6.7 to 8.3 per 100 000 person-years in record-based surveys [1]. Clinical symptoms in PD comprise both motor and nonmotor symptoms. PD patients show slowness of initiation of voluntary movements with progressive reduction in speech (bradykinesia), muscular rigidity, resting tremor, and postural instability. Additionally, it is known that almost 90% of PD patients experience nonmotor symptoms during the course of disease [2]. The spectrum of nonmotor symptoms is also very broad and comprises neuropsychiatric conditions, such as depression, dementia, and hallucinations as well as autonomic, sensory, and REM sleep behavior disorders.

A region-specific selective loss of dopaminergic (DAergic) neuromelanin-containing neurons from the pars compacta of the substantia nigra (SNpc) is the pathological hallmark of PD. However, cell loss in the locuscoeruleus, dorsal nuclei of the vagus, raphe nuclei, nucleusbasalis of Meynert, and some other catecholaminergic brain stem structures including the ventrotegmental area also exists [3]. This neuronal cell loss is accompanied by intraneuronal inclusions: the Lewy body (LB). *α*-synuclein represents one of the most abundant proteins found in LBs, and it may play a pivotal role in the progression of PD. Since the degree of DAergic neuronal loss correlates with the severity of PD, levodopa (L-dopa), a chemical precursor of dopamine (DA) is the most effective drug for the symptomatic treatment of PD. Unfortunately, the clinical efficacy often declines after long-term levodopa replacement therapy (DA replacement therapy; DRT), and additionally, disabling adverse effects appear, most notably motor fluctuation such as the wearing-off or on-off phenomenon and dyskinesia [4]. 

 Nowadays, DA receptor agonists are even regarded as first choice in *de novo* and young PD patients to delay onset of levodopa therapy. They are also used as combination therapy together with levodopa to retard the development of motor complications in advanced stages of PD. DA receptor agonists appear to act by not only direct stimulation of postsynaptic DA receptors but also presynaptic receptors. However, DA receptor agonists may be slightly less potent medicines than levodopa and may be poorly tolerated by older PD patients. Additionally, long-term therapy with traditional ergot DA receptor agonists may result in valvular heart disease [5–8]. A lack of spontaneity or reduced motivation (i.e., anhedonia) is the most troublesome issue in the therapy of advanced stage of PD patients. Medicines with high affinity for the DA receptors potentially improve these symptoms [9, 10]; however, hedonistic dysregulation syndrome or DA dysregulation syndrome (DDS) has emerged as a serious issue in PD with long-term DRT [11]. 

In this paper, we describe the physiology of DA receptors, the characteristics of animal models that have undergone genetic manipulation of DA receptors, the significance of DA receptors stimulants in therapeutic strategies of PD, and the issues of motor and nonmotor complications with long-term treatment.

## 2. Physiological Characterization of DA Receptors

DA is a prototypical slow neurotransmitter that plays significant roles in a variety of not only motor functions but also cognitive, motivational, and neuroendocrine [18]. All members of receptors share a number of structural characteristics such as (1) seven hydrophobic transmembrane stretches, (2) significant amount of amino acid sequence identity between different subfamily within these transmembrane regions and posttranslational modifications such as glycosylation and phosphorylation, and (3) conserved amino acid residues that are involved in interaction of G-protein and in binding agonists [19] (Table 1). On the basis of biochemical, pharmacological, and physiological criteria, DA receptors have been classified into two subfamilies, termed D1 and D2 [20, 21]. Genes encoding members of the DA receptor family are part of a larger superfamily of genes comprising the G protein-coupled superfamily receptors (GPCRs) [12, 19]. G protein-related actions of GPCRs are mediated by a subset of the heteromeric G protein subtypes. In general, G proteins consist of three protein subunits *α*, *β*, and *γ*. The *α*-subunits are functionally classified into several classes such as G_*α*s_, G_*α*i_, G_*α*o_, G_*α*olf_, G_*α*t_, G_*α*q_, and G_*α*12_ and determine actions of GPCRs. Upon ligand binding, G_*α*_ proteins release GDP and newly bind GTP, then *βγ*-complex dissociates from *α*-subunit. Both the *α*-subunit and the *βγ*-complex can transduce the signal to activate a number of effector systems. For example, the activation of G_*α*s_ subunit stimulates adenylate cyclase (AC), whereas the activation of G_*α*i_ subunit inhibits AC. The D1 subfamily, including D1 and D5, are generally coupled to G_*α*s_, and G_*α*olf_ and stimulate the production of cAMP and activate protein kinase A (PKA) [12] (Table 1). The D2 subfamily, including D2, D3, and D4, are coupled to G_*α*i_ and G_*α*o_ and downregulate the production of cAMP via inhibiting AC, resulting in a decrease in PKA activity [12] (Table 1). One of a PKA substrate, DA and cAMP-regulated phosphoprotein 32-kDa (DARPP-32) is known to be involved in DA receptor signaling. DARPP-32 is a multifunctional phosphoprotein and acts as an integrator involved in the modulation of cell signaling in response to multiple neurotransmitters, including DA [22]. Activated DARPP-32 inhibits protein phosphatase 1 (PP1) and results in activation of mitogen-activated protein (MAP) kinases, such as extracellular signal-regulated kinase (ERK) and MAP/ERK kinase (MEK). MAP kinases play a pivotal role in the regulation of synaptic plasticity and have been shown to be signaling intermediates that are involved in the regulation of DA-associated behavior [23]. 

 Activation of D1 receptor/PKA/DARPP-32 signaling cascade leads to the inactivation of PP1 and allows for the activation of MEK and its downstream kinase ERK. D1 DA receptor-mediated ERK activation might involve the behavioral responses to drugs to abuse. Moreover, D1 subfamily can also couple to G_*α*q_ that regulates phospholipase C (PLC) activation. Activated PLC leads to the production of inositol triphosphate (IP_3_) and diacylglycerol and activation of protein kinase C (PKC). PKC increases mobilization of intracellular calcium in response to IP_3_. D1 subfamily agonist SKF-82526 can activate PLC, and this reaction is completely lost in the hippocampal, cortical, and striatal tissues of D5 receptor knockout mice, indicating D5 receptor plays a role for molecular signaling through G_*α*q_ [24, 25]. Lee et al. demonstrated that coactivation of D1 and D2 receptors elevates intracellular calcium with PLC and PKC activation via coupling to the G_*α*q_ pathway [26]. Several studies indicate that D1 and D2 receptors can coexpress in the brain [27, 28]; thus, it is possible that D1/D2 DA receptor heterodimers exist in vivo and regulate G_*α*q_ signaling pathway (Table 1).

D2 subfamily regulate G_*α*i_ and G_*α*o_/PKA/DARPP-32 signaling cascade and also act on ion channels or by triggering the release of intracellular calcium [12]. D2 receptors regulate G_*βγ*_ subunits signaling. This subunit complex activates PLC and produces IP_3_, resulting in increasing the cytoplasmic calcium concentration [29]. G_*βγ*_ subunits can reduce the level of activity of the L/N-type of calcium channels [30]. Through these diverse cAMP- and Ca^2+^-dependent mechanisms, DA influences neuronal activity, synaptic activity, and behavior [31–34]. Importantly, D2 receptor-mediated G_*βγ*_ subunits signaling regulates not only calcium channels but also potassium channels and G protein-coupled inwardly rectifying potassium channels (GIRK) [35–37]. D2 receptors also regulate *β*-arrestin. The scaffolding proteins *β*-arrestin 1 and *β*-arrestin 2 have been traditionally associated with the termination of GPCR signaling and with receptor internalization. Following GPCR activation and their phosphorylation by GPCR kinases (GRKs), *β*-arrestins bind to the receptors to uncouple them from G proteins and participate in the recruitment of the endocytic protein complex, thus leading to an attenuation of GPCR signaling. *β*-arrestin 2 is a signaling intermediate implicated in the cAMP-independent regulation of Akt and glycogen synthase kinase 3 (GSK-3) by DA [13, 38]. Beaulieu et al. demonstrated that D2 DA receptor-mediated Akt/GSK-3 signaling is disrupted in mice lacking *β*-arrestin 2 [38] (Table 1).

The D1 receptor subfamily is expressed in multiple brain regions, including the cortex, hippocampus, amygdala, and most intensively, the striatum, olfactory bulb, and substantia nigra [39]. In the cortex and hippocampus, D1 receptors are expressed in a subpopulation of interneurons [40], but are primarily expressed in pyramidal neurons, with a predominant subcellular localization in the spines of apical dendrites [41]. D5 receptors coexpresses with D1 receptors in cortical pyramidal neurons, and are predominately localized in shafts [41] (Table 1). 

The expression and localization of D2 receptor subfamily have also been investigated at the cellularand subcellular levels [39, 42–46]. There are two major D2 receptor variants that have been termed D2-long (D2L) and D2-short (D2S) [47, 48]. D2L contains an additional 29 amino acids in the third cytoplasmic loop. D2L and D2S expressed mainly postsynaptically and presynaptically, respectively. D2S might function as autoreceptor that decreases DA release, resulting in decreased locomotor activity; however, activation of postsynaptic D2 receptors stimulates locomotion. D2 receptors, the predominant subtype of thisclass, are expressed in the pituitary gland and basal ganglia (striatum and substantia nigra) and localized in both pre- and postsynaptic structures. Presynaptically, D2 receptors areassociated with both forebrain projecting DAergic afferents and glutamatergic terminalsin the striatum and prefrontal cortex (PFC). Postsynaptically, D2 receptors are concentrated inshafts and spines of both cortical pyramidal neurons and striatopallidal neurons. While both D1 and D2 receptors are abundant in the striatum, the expression pattern of D1 and D2 receptors in the axon terminals, dendrites, and spines are obviously different by electron microscopic analysis [42]. These findings indicate segregated circuit via D1 and D2 receptors in the striatum. D3 receptors are expressed in the olfactory tubercle, nucleus accumbens, striatum, and substantia nigra. Importantly, D3 receptors are also found in limbic system such as hippocampus, septum, or mammillary nuclei of the hypothalamus. The D4 receptors, known to have an unusually high affinity for the atypicalneuroleptic clozapine, are localized in thefrontal cortex, medulla, amygdala, hypothalamus, mesencephalon, and nucleusaccumbens. It has been shown that lower level of D4 receptor expression is detected in the basal ganglia. In the rat central nervous system, the relative abundance of the DA receptors is D1 > D2 > D3 > D5 > D4 [49]. 

Consistent with the degeneration and loss of DA neurons in PD, the density of postsynaptic DA receptors increases in association with developing DA denervation supersensitivity. In 1970s and 1980s, many reports have indicated that D1 and D2 receptors are increased in the untreated PD using radioisotope-labeled high-affinity ligands and return to normal level upon levodopa therapy [50–52]. Interestingly, GIRK2 knockout mice exhibited hyperactivity, which was inhibited by the D1/D5 receptor antagonist SCH-23390 [53]. SCH-23390, D1 agonists SKF-38393, and SKF-81297 can reversibly inhibit GIRK channel, and prior application of a GIRK channel blocker eliminated the depolarizing effect of D1 agonists [54, 55]. Witkowski et al. conclude that activation of D1DA receptors may lead to inhibition of GIRK channel currents [55]. A selective D3 agonist PD-128907 is one of the most useful compounds for investigating behavioral change. PD-128907 reduced spontaneous locomotor activity in a novel environment [56, 57]. Eticlopride and raclopride are selective D2 receptor antagonists. Eticlopride has been shown to robustly block the reinforcing effects of cocaine [58–60]. Increased densities of D1 and D2 DA receptors may account for the clinical sensitivity to DA agonists in the PD patients, especially in early stage. Scherfler et al. demonstrated that raclopride-binding potential is significantly decreased in striatal, thalamic, and cortical areas (temporal, orbitofrontal, and parietal cortex) in *parkin*-linked PD patients compared with young onset PD patients, matched for age, severity, and duration [61]. They discussed that the decrease in D2 binding found in *parkin*-linked PD compared with young onset PD patients could be a direct consequence of the *parkin* genetic defect itself or a greater susceptibility to receptor downregulation following long-term DAergic agent exposure. Boileau et al. performed positron emission tomography with D3 receptor preferring ligand propyl-hexahydro-naphtho-oxazin (PHNO) in brain of nondepressed, nondemented, DAergic drug-naïve patients with early-stage PD [62]. They demonstrated that decreased binding ratio correlated with motor deficits and lowered mood. These results indicate that DA receptors and their binding potential may gradually decrease in the progression of PD.

It has long been suggested that DA dysfunction plays a major role in the pathogenesis of schizophrenia. Antipsychotics such as chlorpromazine and haloperidol act primarily as D2 receptor antagonists [63] (Table 1). These D2 receptor blockades are effective in attenuating positive symptoms of schizophrenia (e.g., hallucinations and delusions) associated with acute episodes as well as preventing psychotic relapse. Wong et al. demonstrated that D2 receptor density in the caudate nucleus is elevated in drug-naïve schizophrenia patients with positron emission tomography (PET) study [64]. Breier et al. also showed that patients with schizophrenia compared with healthy volunteers had significantly greater amphetamine-related reductions in radioligand [^11^C] raclopride-specific binding ratio with PET [65]. This result indicates that schizophrenia is associated with elevated amphetamine-induced synaptic DA concentrations [65] (Table 1). Studies also showed that increased striatal DA synthesis capacity in unmedicated schizophrenia patients [66, 67]. These results indicated that the pool of releasable DA is increased in patients with schizophrenic psychosis. Kramer et al. demonstrated that a selective D4 receptor agonist L-745870 is ineffective in patients with schizophrenia [68]. 

## 3. Genetic Manipulation of DA Receptors and Pathophysiology of PD

The facts regarding the contribution of D1- and D2-type DA receptors in the genesis of the behavioral and neurochemical Parkinsonian phenotype have been provided byclassic pharmacological approaches. Recently, genetically modified mice of DA receptors are also clarifying the significance of specific effects of DA-related neuronal physiology and pathophysiology. 

In 1994, Xu et al. and Drago et al. generated D1 receptor-deficient mice [69, 70]. Xu et al. demonstrated that these mice also appear to exhibit a general behavioral hyperactivity during both phases of light-dark cycle [69]; however, Drago et al. described that the locomotor activity of knockout mice did not differ significantly from that of normal control except for displaying a significant decrease in rearing behavior [70]. Test for akinesia were normal. Moreover, Gantois et al. used a *Cre*/*Lox *transgenic approach to generate ananimal model in which D1 receptor-expressing cells are progressively ablated in the postnatal brain [71]. Whereas no differences in locomotor activity were found between the mutated mice and control, mutant showed hyperactivity in a novel environment. Abnormal oral behaviors such as chewing and sifting, limb-clasping dystonic posture, and spontaneous seizure are also found in mutant mice. Increased locomotor activity of D1 receptor-deficient mice is cancelled with a D1/D5 receptor antagonist SCH 23390, indicating that D1 and D5 receptors can exert distinct and complex physiological actions in locomotor activity [72]. 

In contrast to disruption of D1 receptor subfamily, spontaneous PD-like locomotor impairment is found in D2 receptor subfamily knockout mice. D2 receptor-deficient mice exhibit significantly reduced spontaneous movements in behavioral tests [73]. However, Kelly et al. demonstrated that striatal tissue content of monoamines and their metabolites from C57BL/6 congenic strain of D2 receptor mutant mice did not differ from those of wild type, and they found no evidence for supersensitive D1, D3, or D4 DA receptors in the D2 receptor knockout mice [74]. Recently, Tinsley et al. showed that LB-like cytoplasmic inclusions containing *α*-synuclein and ubiquitin were present in substantia nigra neurons of older D2 receptor knockout mice (>18 months old) [75]. Diffuse cytosolic *α*-synuclein immunoreactivity in nigral neurons increased with age in both wild-type and knockout mice, most likely because of redistribution of *α*-synuclein from striatal terminals to substantia nigra cell bodies. Gene and protein expression studies showed endoplasmic reticulum (ER) stress and changes in trafficking and autophagic pathways, indicating that these changes were accompanied by a loss of DA terminals in the dorsal striatum [75]. Wang et al. showed behavioral alteration in the D2L receptor-deficient mice [76]. The knockout mice display reduced locomotor activity and rearing behavior and reduced sensitivity to haloperidol-induced catalepsy. These results indicate that D2L might have a bigger impact on certain types of motor functions, and blockade of D2L might contribute more than blockade of D2S to the extrapyramidal side effects (or parkinsonism) that are commonly associated with typical antipsychotic drugs [76]. In addition, D2 receptor plays a pivotal role in the striatal processing of motor information received from the cortex in the generation of striatal synaptic plasticity. Whereas tetanic stimulation of corticostriatal fibers produced long-term potentiation (LTP) in the D2 receptor-null mice, long-term depression (LTD) is usually recorded in wild-type mice [77]. The LTP in knockout mice is blocked by an NMDA receptor antagonist, indicating that D2 receptor is involved in the formation of striatal LTD and exerts a negative control in the expression of an NMDA-mediated long-term LTP at corticostriatal synapses. Adenosine A2A receptors (A2AR) are known to coexpress D2 receptors in striatopallidal neurons, and A2AR-D2 receptor interaction modulates DA-mediated signaling [78]. A2AR selective antagonist, (E)-1,3-diethyl-8-(3,4-dimethoxystyryl)-7-methyl-3,7-dihydro-1Hpurine-2,6-dione (KW-6002, Istradefylline), exhibits anti-Parkinsonian activities [79]. Aoyama et al. demonstrated that locomotor impairment can be relieved by KW-6002 treatment in D2 receptor knockout mice [80]. Furthermore, the level of the expression of enkephalin and substance P is elevated to normal levels after A2AR antagonist treatment. These results show that A2AR and D2 receptor have antagonistic and independent activities in controlling neuronal and motor functions in the basal ganglia [80].

Although D3 receptor-deficient mice do not exhibit parkinsonism, it is quite important to elucidate specific behavioral alteration in the knockout mice for understanding clinical features of pharmacotherapeutic DA receptor agonists in the treatment of PD. D3 mutant mice exhibit hyperactivity in novel and exploratory environment and rearing behavior [81–83]. These mutant mice demonstrated that both D1- and D2-receptor-binding sites are present in the dorsal and ventral striatum and the distributions and the densities of both binding sites in the mutants and controls are qualitatively and quantitatively similar [82]. Importantly, hyperactivity of mutant mice is caused by response to combinations of D1 and D2 receptor subfamily agonists, cocaine, and amphetamine. Carta et al. also showed that acute administration of cocaine resulted in increasing mRNA level of *c-fos* and *dynorphin* in the dorsal and ventral striatum of D3 receptor knockout mice, indicating D3 receptor plays a role on gene regulation in the DA system [84]. Moreover, Schmauss found that c-*fos *mRNA levels expressed in response to D1 agonist or methamphetamine administration is significantly blunted in D3 receptor-deficient (and also D2 receptor-deficient) mice [85]. D3 receptor mutants exhibit deficits in their spatial working memory, and methamphetamine pretreatment does not rescue this memory deficit of D3 mutants [85, 86]. These results indicate that the constitutive inactivation of D3 receptors leads to a decrease in agonist-promoted D1 receptor activity. D'Agata et al. have investigated *parkin* expression profile in D3 knockout mice [87]. *parkin* is known as one of the causative genes of autosomal recessive juvenile Parkinson's disease. Parkin protein has an E3 ubiquitin-ligase activity, and loss of parkin function may result in accumulation of unnecessary molecules that lead to the degeneration of neurons. Real-time PCR analysis showed a different quantitative expression of *parkin* gene in mutant compared to control mice. Furthermore, immunoreactivity of parkin showed a higher intensity in D3 receptor knockout mice compared to wild type by Western blot analysis using parkin mouse monoclonal antibody [87]. Karasinska et al. generated mice lacking both D1 and D3 receptors and investigated psychostimulant-induced behavior [88]. Administration of cocaine increased locomotor activity in wild-type and D3 knockout mice, failed to stimulate activityin D1 knockout mice, and reduced activity in D1/D3 knockout mice. Karasinska et al. discussed the significance of expression level of phosphorylated cAMP-responsive element-binding protein (pCREB) in the striatum. CREB is activated by phosphorylation in striatal regions following DA receptor activation. Striatal pCREB levels following acute cocaine were increased in D3 mutant mice and decreased in D1 and D1/D3 mutant mice [88]. The change of locomotor activity of D3 mutant mice is controversial. Jung et al. demonstrated that the activity of D2/D3 double mutants is significantly reduced not only when compared to wild type but also when compared to single D2 receptor mutants [89]. They indicated that a relatively long observation period for locomotor activity is needed in D3 mutant mice because of their rapidly habituating hyperactivity [89]. 

D4 knockout mice show significantly reduced exploration behavior and rearing activity [90, 91]. Extent of improvement of locomotor activity is dramatically increased in D4 receptor-deficient mice than that of wild type of littermate following the administration of ethanol, cocaine, and amphetamine, indicating that knockout mice are more responsive to the locomotor stimulants than wild type [90]. Falzone et al. described that the absence of D4 receptor increases avoidance behavior to unconditioned stimuli and does not impair behavioral reactions to fear-conditioned stimuli in two different approach/avoidance conflict paradigms [92]. These results indicate that D4 receptor could play a pivotal role in the DAergic modulation of cortical signals triggered by environmental stimuli, because D4 receptor is physiologically expressed at highest levels in the prefrontal cortex and is the predominant D2-like receptor localized in this brain area (Table 1). 

## 4. Treatment for PD with DA Receptor Agonists

As a result of significant advances in experimental therapeutics *in vitro* and *in vivo*, progression of treatment for PD has been made, and many promising therapies are emerging. L-dopa is classical, but it remains the most potent medication for controlling PD symptoms [96]. Unfortunately, despite adjustments of the timing and dose frequency of L-dopa, motor fluctuation (such as wearing-off and on-off phenomena) and involuntary movements (such as dyskinesia and dystonia) could tend to appear in the PD patients who are treated long duration of L-dopa medication. There is ongoing debate as to when it is most appropriate to initiate L-dopa therapy in the course of PD. Most physicians and neurologists pay attention to timing for starting of L-dopa therapy in order to delay the onset of related motor complications. DA receptor agonists are now known to have beneficial effects to delay onset adverse effects of L-dopa and recommended as first choice treatment in the relatively young-onset PD patients. 

DA receptor agonists exert their pharmacologic effect by directly activating DA receptors, bypassing the presynaptic synthesis of DA (Table 2). In general, half-lives of agonists are remarkably longer than L-dopa; therefore, they can produce more persistent period of DA receptor stimulation than L-dopa. In addition, metabolites of receptor agonists do not generate free radicals that are toxic on DA neurons [97]. DA receptor agonists also have been suggested to be neuroprotective acting as free-radical scavengers, reducing DA synthesis, DA release and metabolism via activating presynaptic autoreceptors, ameliorate excitotoxicity by suppressing subthalamic nucleus overactivity, and exert antiapoptotic effects (review in [98–101]). Experimental (as described above) and clinical studies have provided evidence that activation of the D2 receptors (D2 and D3 receptor) is beneficial for treatment of PD; however, pharmaceutical receptor agonists also stimulate D1 receptor to some extent.

 DA receptor agonists are classified by their chemical structural formula as ergoline derivatives including bromocriptine, pergolide, cabergoline, lisuride, and *α*-dihydroergocriptine (DHEC) and nonergoline derivatives including pramipexole, ropinirole, rotigotine, and apomorphine. Ergolines are derivative of ergot alkaloids that have a longer history in PD therapy and are as effective as nonergolines. Nonergolines have been developed later in the hope that they could provide the benefits of the ergolines without their adverse effects.

### 4.1. Bromocriptine (Parlodel and Cycloset)

Bromocriptine is the first therapeutic DA receptor agonist (Table 2). It has been shown to protect mice against 6-hydoxydopamine (6-OHDA) and can scavenge methamphetamine-induced free radicals [102–104]. The pharmacological neuroprotective effects of bromocriptine comprise not only D2 receptor agonist but also antioxidant free-radical scavenger. It also exerted a neuroprotective effect against glutamate-induced cytotoxicity in rat mesencephalic and cortical neurons [105, 106]. Kihara et al. indicated that bromocriptine could upregulate Bcl-2 expression via phosphatidylinositol 3 kinase cascade [106].

### 4.2. Pergolide (Permax)

Pergolide is rapidly absorbed following oral dosing, reaching peak plasma concentrations within 2-3 hours, and it is completely eliminated within 4-5 days (review in [107]) (Table 2). Pergolide has a long half-life of about 21 hours. This has interesting implications, as it should produce a more physiological or continuous stimulation of DA receptors, avoiding or delaying the induction of dyskinesia [107]. Similar to bromocriptine, pergolide also has been shown to have the neuroprotective effect *in vitro* and* in vivo * [97, 108, 109]. Long-term oral administration of pergolide results in preventing age-related diminution of DA cell bodies in the rat substantia nigra [108]. Asanuma et al. also reported that repeated pretreatment with pergolide significantly protected against 6-OHDA-induced reduction in striatal DA content [109]. The neuroprotective effect of pergolide has been shown to be mediated by free-radical scavenging activity [110].

### 4.3. Cabergoline (Cabaser and Dostinex)

Cabergoline has a very long elimination half-life of 65–110 hours (Table 2). Arai et al. showed that combined therapy with low doses of L-dopa and cabergoline is beneficial for treating patients with PD [111]. This combination is highly effective in controlling motor disability without inducing dyskinesia in 1-methyl-4-phenyl-1,2,3,6-tetrahydropyridine- (MPTP-) lesioned Parkinsonian cynomolgus monkeys [111]. Administration of cabergoline also attenuates 6-OHDA-indced DAergic neuronal loss in mice [112]. Daily administration of cabergoline to mice significantly increased striatal glutathione (GSH) levels by activation of RNA expression of GSH-related enzymes, indicating scavenging free radicals by activation of the GSH system [112]. Cabergoline is also an inducer of glial cell line-derived neurotrophic factor (GDNF) that may have neuroprotective and neurorestorative properties in DAergic nigral neurons [113].

### 4.4. Lisuride (Dopergin, Proclacam, and Revanil)

Lisuride is used to lower prolactin and to prevent migraine attacks. It has not only a D2 receptor agonist properties but also has 5-hydroxytryptamine (5-HT and serotonin) agonist activity. Terminal half-life for elimination of lisuride from the plasma is around 2 hours, which is shorter than most other DA agonists (Table 2). Lisuride has solubility properties similar to apomorphine and therefore, can be given subcutaneously and intravenously. Nomoto et al. studied the effects of lisuride in the dermal application on MPTP-treated common marmosets and on 5 patients with PD [114]. The agent relieved akinesia and Parkinsonism of MPTP-treated monkeys and increased the duration of “on” period in the patient of PD. The other important feature of lisuride is that it has no affinity to 5-HT_2B_ receptors [115, 116]. Therefore, lisuride might not a causative agent for valvular heart disease and fibrotic reactions that could be related to other ergot DA receptor agonists described below.

### 4.5. ***α***-dihydroergocriptine (DHEC)

DHEC is a dihydro-derivative of ergocryptine acting as a D2agonist and a partial D1 agonist. Therefore, DHEC has a pharmacodynamic profile quite comparable to that of bromocriptine. DHEC improves the symptoms and neuronal degeneration of the MPTP-treated monkey model of PD [117, 118]. In cellular models, DHEC protected cultured granule cells against age-dependent neuronal cell death and increased the survival of DAergic cells when coadministered with L-dopa and DA [119, 120]. 

### 4.6. Pramipexole (Mirapex, Mirapexin, and Sifrol)

Pramipexole is one of a representative nonergot DA receptor agonist that has a potent activity to D2 and D3 receptors [121] (Table 2). It has been shown to be a safe and effective drug when used as monotherapy in early-to-moderate stages and adjunct therapy with L-dopa in advanced stage of PD [122–124]. Importantly, pramipexole reduced not only motor symptoms of PD but also significantly reduced anhedonia in patients who had associated depression [124]. Recent studies exhibited that pramipexole improves both subjective symptoms of restless legs syndrome (RLS), mainly characterized by discomfort at rest and urge to move focused on the legs, and objective sleep parameters [125, 126]. Zou et al. showed that the neuroprotective effect of pramipexole is the result of antioxidant property [127, 128]. Pramipexole also interferes with the activation of caspase-mediated cell death pathways in rotenone- and MPTP-administered human neuroblastoma cell line SH-SY5Y [129, 130]. Interestingly, The protective effect was not prevented by DA receptor blockade using sulpiride or clozapine, indicating that pramipexole acts by other protective mechanism that is not dependent on DA receptor occupation [130]. Izumi et al. revealed that pramipexole protects DAergic neurons from glutamate cytotoxicity by the reduction of intracellular DA content [131]. Ramirez et al. reported that pramipexole plays a role for D3 receptor-mediated neuroprotection against MPTP-induced neurotoxicity with D3 receptor knockout mice [132]. Furthermore, the DA D3 receptor selective antagonist A-437203 partially inhibited the neuroprotective effect of pramipexole in D3 receptor expressing mice but not in knockout mice. These results indicate that D3-dependent and D3-independent pramipexole-mediated neuroprotection seem to act against MPTP neurotoxicity.

### 4.7. Ropinirole (Requip, Ropark, Adartrel, and Ropinotergotirole)

Ropinirole is a nonergoline agonist and has a high affinity for D2 and D3 DA receptors (Table 2). It has been also demonstrated to be effective in early and advanced PD [133–135]. Monotherapy is also effective in early stage of PD with resting tremor [136]. Similar to pramipexole, ropinirole is also well tolerated in patients with RLS [137, 138]. Recent report indicates that ropiniroleis more effective for acute therapeutic efficacy with regard to periodic leg movement in RLS than gabapentin [139]. In animal model of PD, ropinirole reverses motor and behavioral deficits induced by MPTP in marmosets [140] and protects striatal DAergic neurons against 6-OHDA in mice via increased GSH, catalase, and superoxide dismutase [141]. Matsukawa et al. revealed that lentiviral overexpression of both D2 and D3 receptor in 6-OHDA-administered rats results in the enhancement of efficacy of ropinirole for motor symptoms [142]. 

### 4.8. Rotigotine (Neupro)

Rotigotine is a unique nonergoline DA receptor agonist for D1 through D5 receptors, particularly in the highest affinity for D3 receptor [93] (Table 2). It has been formulated for transdermal delivery that ensures continuous drug release and stable plasma concentration over a period of 24 hours [143]. Various formulations of L-dopa have been developed to delay levodopa degradation in peripheral tissues and prolong its half-life, and thus minimize plasma peaks and troughs of drugs. In addition to their symptomatic benefits and in delaying the initiation of L-dopa therapy, agonists have also been used to provide more continuous DAergic stimulation. Randomized, blind, controlled trials of transdermal rotigotine revealed efficacy and safety in the treatment of early and advanced PD [144, 145]. Transdermal rotigotine is also effective in moderate-to-severe RLS [146, 147]. In rodent and primate PD models, rotigotine treatment demonstrated its protective effects from MPTP toxicity and efficacy of behavioral symptoms improvement [148, 149].

### 4.9. Piribedil (Trivastal, Pronoran, Trastal, and Trivastan)

Piribedil is a centrally acting DA agonist with an affinity for D2 and D3 receptors [150, 151] (Table 2). Ziegler et al. demonstrated that a 6-month oral administration of piribedil in combination with L-dopa is well tolerated and significantly improves motor symptoms compared with placebo in PD nonfluctuating patients [152]. In 6-OHDA-induced rat animal models, combination therapy with piribedil and L-dopa can improve cognitive functions while reducing risk of motor complications [153]. Moreover, piribedil monotherapy is shown to be effective for the improvement of motor symptoms and safe with early stage of PD [154]. A recent study of a single-dose placebo-controlled, randomized, double-blind study indicates that a novel orodispersible formulation of the piribedil (S90049) is promising to provide relief on motor signs and aborting “off” episodes of PD [155]. Smith et al. generated common marmoset PD model induced by MPTP and treated with L-dopa to improve motor deficits but with mild dyskinesia [156]. Then, switching treatment from L-dopa to piribedil resulted in decreasing dyskinesia without symptomatic deterioration, indicating that piribedil could suppress dyskinesia induced by long-term treatment with L-dopa. 

### 4.10. Apomorphine (Apokyn, Ixense, Spontane, and Uprima)

Apomorphine is a nonergoline and a nonselective DA receptor agonist [157] (Table 2). Historically, it was the first DA receptor agonist used for treating PD since 1950s. It is first known since 1845 for its emetic properties, and it has been utilized for different clinical situations, such as analgesia, insomnia, alcohol dependence, schizophrenia, and erectile dysfunction. Since apomorphine has a rapid absorption and a short half-life, the best mode of administration is subcutaneous intermittent injection or continuous infusion. Continuous infusion can be used as monotherapy or in concomitance with extremely low doses of L-dopa. Apomorphine also can reduce “off” periods, improve motor symptoms of PD, and suppress dyskinesia (reviewed in [158]). Like other DA receptor agonists, there are several reports describing the neuroprotective potential of apomorphine in animal models. Pretreatment with apomorphine administered subcutaneously in mice protects against MPTP-induced loss of nigrostriatal DA neurons as indicated by striatal DA content [159]. Similarly, Battaglia et al. revealed that continuous subcutaneous infusion of apomorphine rescues loss of striatal immunoreactivity for tyrosine hydroxylase and DA transporter in MPTP-injected mice [160]. Apomorphine treatment significantly attenuated the 6-OHDA-induced striatal DA depletion, and dihydroxyphenyl acetic acid (DOPAC)/DA ratios, a neurochemical indicator for neuroprotection, were normalized [161]. The neuroprotective effect of apomorphine could be a consequence of antioxidant activity, potent iron chelating action, inhibition of lipid peroxidation, induction of neurotrophic factors, and anti-inflammatory effects [159, 162–166].

### 4.11. Adverse Effects Associated with DA Receptor Agonists

Despite the fact that DA receptor agonists have the advantage of providing symptom control without increasing amount of L-dopa and dyskinesia or wearing-off effect that occurs with long-term L-dopa treatment, physicians and neurologists noticed several PD patients treated with DA receptor agonists developed critical side effects. Fibrotic valvular heart disease and DRT-induced pathological behaviors (DDS) are representative features of agonists. Physicians must consider balance the benefits of continued therapy with agonists against these possible risks. Further investigations are necessary to assist with treatment decisions.

### 4.12. Fibrotic Valvular Heart Disease

Fibrotic heart disease is mainly induced by ergoline derivatives. Pritchett et al. first described three pergolide-treated PD patients who developed unexplained tricuspid regurgitation in 2002 [167]. The other cases that indicate other ergot DA receptor agonists such as cabergoline and bromocriptine seem to be causative agents [168, 169]. No patients given lisuride have developed valvulopathy. Several studies of echocardiographic prevalence have shown moderate to severe valvular disease in about ~31% of patients taking pergolide and in about ~69% taking cabergoline [8, 170–173]. These studies have not performed echocardiographic analysis before starting ergoline DA receptor agonists; therefore, preexisting valvular heart disease cannot be excluded. Schade et al. investigated population-based cohort study with data from the United Kingdom General Practice Research Database [174]. After excluding subjects that have preexisting valvular heart disease, of 31 cases from comprising 11,417 patients who had received anti-Parkinsonian drugs newly developed valvular regurgitation. 31 cases from comprising 11,417 patients who had received anti-Parkinsonian drugs newly developed valvular regurgitation. 12 patients have been taking pergolide or cabergoline. The rate of cardiac-valve regurgitation is increased with current use of pergolide (adjusted incidence-rate ratio, 7.1) and cabergoline (4.9) [174]. 

It remains controversial whether a high daily dose of prescribing agonists or cumulative dose of drugs is true risk for prevalence of valvulopathy. Van Camp et al. discussed that 5 mg or high daily dose of pergolide administration promotes heightened risk for development heart disease; however, this result was not statistically significant [175]. Other study indicates that 3 mg or high daily dose of pergolide and cabergoline raised risk although it is still increased in patients who are received 3 mg or less of either drugs [174]. On the other hand, the risk of valvulopathy might be small in patients treated with low dose of agonists. Kim et al. indicated no significant difference in the frequency of valvulopathy in the subjects receiving a mean daily dose of 1.13 mg of pergolide for mean duration of 53 months compared with controls [171]. Yamamoto et al. also showed that there was no significant high risk in the frequency of moderate-to-severe valvular regurgitation in the PD patients receiving a mean daily dose of 1.4 mg of pergolide [170]. Few studies showed natural history of pergolide/cabergoline-induced valvular heart disease. Pinero et al. described a patient who developed severe mitral valve regurgitation after 4 months treatment with cabergoline [176]. Moreover, some of these drug-induced valvulopathy case showed partial improvement after stopping pergolide/cabergoline treatment [172, 175]. 

 The pathogenesis of DA receptor agonist-induced valvulopathy is associated with 5-HT_2_ and 5-HT_2_ receptor-mediated signaling. 5-HT_2_ has a significant mitogenic effect on smooth muscle cells and fibroblast with upregulation of transforming growth factor *β* (TGF-*β*) [177–179]. 5-HT_2B_ receptor is abundant on cardiac valves and ergoline DA receptor agonists could stimulate these receptors, resulting in mitogenesis and thickening of valves [180]. Moreover, 5-HT transporter- (5-HTT-) deficient mice showed marked valvular fibrosis and 5-HT_2_ administration results in downregulating gene expression of 5-HTT [181, 182]. These results indicate that modulation of 5-HTT expression also plays a pivotal role for pathogenesis of valvulopathy. 

 Whereas pergolide has been withdrawn from the US market, most ergoline DA receptor agonists still remains in use in the rest of the world. In many countries, guidelines recommended relatively low daily dose of pergolide/cabergoline to decrease the prevalence of valvular heart disease. It is also recommended to perform echocardiography before starting treatment and regularly thereafter.

### 4.13. DRT-Induced Pathological Behaviors (DDS)

Anti Parkinsonian drug-induced nonmotor complications have been significant problem in PD patients. Giovannoni et al. described four patients and proposed diagnostic criteria for DDS as follows [11]: L-dopa responsive PD; need for excessive dose of DRT to relieve symptoms, drug hoarding and seeking, impairment of social or occupational functioning, development of affective syndrome related to DRT, development of a withdrawal state on reducing the level of DRT, and duration of disturbance of at least six months. DDS observed in PD patients could be characterized as impulsive, compulsive, and stereotyped behavior such as punding, pathologicalInternet use, compulsive skin picking, pathological (compulsive) gambling, compulsive buying, binge eating, and hypersexuality. 

 Punding behavior is characterized as compulsive hobbyism. An intense inappropriate and unproductive fascination for common objects with repetitive meaningless movements such as endless computer use, cleaning and tidying, gardening, collecting, and sorting common objects [183]. PD patients with punding are found to take higher dose of DRT and to endure more severe dyskinesia than those who do not pund [184]. Reduction of amount of DRT and/or treatment with antidepressant drugs such as selective serotonergic reuptake inhibitors are effective for improvement of punding.

 Pathological gambling and shopping are failure to resist compulsive gambling/buying that are associated with adjunctive DA agonist therapy but not with DA agonist monotherapy [185, 186]. Voon et al. estimated that lifetime prevalence of pathologic gambling was 3.4% and on any DA agonist was 7.2% [185]. It was associated with earlier PD onset and with DA agonists but not with agonist subtype or doses. Reduction or stopping of these drugs improves this pathological behavior. 

 Hypersexuality is defined as a preoccupation with sexual feelings and thoughts, nymphomania or satyriasis may occur in 2%–4% of the patients [185, 187, 188]. It is associated with DA receptor agonists; however, it has been reported in patients on L-dopa monotherapy and after subthalamic deep brain stimulation [187–189]. Although this abnormal behavior easily disrupts social and marital life, it is rather refractory and often difficult to improve after ceasing the DA receptor agonists and/or adding antipsychotic drugs. 

 Potential mechanism for DDS associated with DA receptor agonists are not fully understood. DA is not only implicated in voluntary movement control but also plays a significant role in the brain's reward system, learning and decision making mechanism, and the modulation of behaviors. The ventral part of striatum receives input from the amygdala, hippocampus, and anterior cingulate cortex, whereas the dorsal part outputs information of motor circuits [190, 191]. In PD, striatal DA deficiency results in general striatal dysregulation, both motor (dorsal part) and nonmotor (ventral part) symptoms. Exaggerated amplification of a normal limbic input in the dorsal part of striatum could result in stereotyped motor output (punding). Overactivation of the ventral part of the striatum is suggested to be associated with increased compulsive and stereotyped behaviors. 

## 5. Concluding Remarks

In the late 20th century, many outstanding studies of the basic genetic and structural features of DA receptors have brought about remarkable progression for therapeutic strategy of several disorders caused by malfunction of DA receptors and DA signaling such as PD and schizophrenia. More recently, many studies have revealed information on the regulatory and signaling mechanisms that are involved in DA receptor signaling. Signaling events that are caused by the activation of each DA receptor are significantly complex and these mechanisms depend on the many molecules such as ligands and kinases. On the other hand, further studies are needed to clarify the characteristics of D3, D4, and D5 receptors. Discovery of new features of these receptors may provide better understanding roles of DA outside of the basal ganglia and give rise to innovations of pharmacotherapeutic strategy of PD treatment. Nonmotor complications and DDS are still major issues for management of the patients with PD. Further investigations of normal and abnormal DA receptor signaling *in vivo* will continue to evolve in exciting ways in the future.

##  Conflict of Interests

The authors declare no conflict of interests.

## Figures and Tables

**Table 1:** Dopamine receptor family.

	D1 subfamily		D2 subfamily		
	D1 (MIM: 126449) (Gene ID: 1812)	D5 (MIM: 126453) (Gene ID: 1816)	D2 (MIM: 126450) (Gene ID: 1813)	D3 (MIM: 126451) (Gene ID: 1814)	D4 (MIM: 126452) (Gene ID: 1815)

Locus (human)	5q35.1	4p16.1	11q23	3q13.3	11p15.5

Amino acids Molecular weight	446 49, 162	477 52,820	443 (D2L)/414 (D2S) 50, 489/47,216	400 44,064	419 43,771
G-protein coupling	G*α*s, G*α*olf	G*α*s, G*α*q	G*α*i, G*α*o, G*βγ*	G*α*i, G*α*o, G*βγ*	G*α*i, G*α*o, G*βγ*

Signaling molecules	AC↑, cAMP, PKA, DARPP-32, ERK, MEK GIRK?	PLC, PKC, IP_3_	AC↓, cAMP, PKA, DARPP-32		
			L/N-type calcium channel		
			GIRK		
			*β*-arrestin2, Akt, GSK-3 (D2, D3)		

Localization	Striatum Nucleus accumbens Olfactory bulb Substantia nigra Amygdala Cortex Hippocampus	Cortex Substantia nigra Hypothalamus Hippocampus	Striatum Nucleus accumbens Ventral tegmental area Substantia nigra Olfactory bulb	Olfactory bulb Nucleus accumbens Striatum Substantia nigra Hippocampus Hypothalamus	Frontal cortex Amygdala Hypothalamus Mesencephalon Nucleus accumbens.

Selective agonists	SKF-38393 SKF-81297 Fenoldopam (SKF-82526)	—	Bromocriptine Pergolide Cabergoline Ropinirole	7-OH-DPAT Pramipexole Rotigotine PD-128907	A-412997 ABT-670 PD-168077

Selective antagonists	SCH-23390 SCH-39166 SKF-83566	—	Haloperidol Raclopride Sulpiride Spiperone Risperidone	Nafadotride GR-103691 GR-218231 SB-277011A NGB-2904 PG-01037 ABT-127	A-381393 FAUC213 L-745870 L-750667

Abbreviations: AC, adenylatecyclase; cAMP, cyclic AMP; PKA, protein kinase A; DARPP-32, 32-kDa DA and cAMP-regulated phosphoprotein; ERK, extracellular signal-regulated kinase; MEK, mitogen-activated protein/ERK kinase; GIRK, G protein-coupled inwardly rectifying potassium channels; PLC, phospholipase C; PKC, protein kinase C; IP_3_, inositol triphosphate; GSK-3, glycogen synthase kinase 3; 7-OH-DPAT, 7-hydroxy-*N*,*N*-dipropyl-2-aminotetralin.

This table is complied from information in several review reports and articles [12–17]. Data of amino acids and molecular weight of each receptor are referred from NCBI, build 37.2 (D1; NP_000785.1, D2L; NP_000786.1, D2S; NP_057658.2, D3; NP_000787.2, D4; NP_000788.2, D5; NP_000789.1).

**Table 2:** Pharmacological features of ergoline DA agonists.

	Ergoline derivatives				Nonergoline derivatives				
	Bromocriptine	Pergolide	Cabergoline	Lisuride^#^	Pramipexole	Ropinirole	Rotigotine	Piribedil	Apomorphine^§^

T_max_ (hr)	2.7	1~3	1.9	0.4	1.4	1.6	15–18	1	0.17~1
C_max_ (ng/ml)	0.47 (5 mg)	1.8 (138 *μ*g)	0.078 (2 mg)	0.63 (0.4 mg)	0.58 (0.2 mg)	0.68 (0.4 mg)	—	—	—
t_1/2 _(hr)	15 (8~20)	15~42	43	0.73	6.4	5	5~7	6.9	0.67
Protein binding (%)	90~96	90	62	15	90~93	35~42	92	—	10

*pK*_*i*_ D1	6.16	6.47	6.67	7.19	<5	<5	7.08^‡^	<5	6.43
*pK*_*i*_ D2	8.30, 7.83	7.50, 7.59	9.21, 9.02	9.47, 9.18	6.02, 5.77	6.17, 6.03	7.87^‡^	6.88, 6.76	7.46, 7.08
*pK*_*i*_ D3	8.17	8.26	9.10	9.55	7.98	7.43	9.15^‡^	6.63	7.59
*pK*_*i*_ D4	6.43	7.23	7.25	8.34	6.89	6.07	8.41, 7.82, 8.22^‡^	6.52	8.36
*pK*_*i*_ D5	6.27	7.48	7.65	8.45	<5	<5	8.27^‡^	<5	7.83
5-HT receptors	1D > 1A > 2B	1A > 2B > 2A	2B > 2A > 1A	1A > 1D > 2B	1A > 1B > 1D	1A > 1D > 2B	1A ≫ 1D ≫ 2B	1A > 2B	2C > 1A > 2A
Adrenoreceptors	*α*1D > 1B > 1A	*α*2B > 2A > 2C	*α*2A > 2C > 1B	*α*2A > 2B = 2C	*α*2B > 2A	*α*2B > 2C > 2A	*α*2B > 2C > 1A	*α*2C > 2A > 1D	*α*2C > 1D > 2B

Abbreviations: T_max_, maximum drug concentration time; C_max_, maximum drug concentration; t_1/2_, half-life; All pharmacokinetic data are referred to package inserts of each medicines and/or Website of DailyMed (http://dailymed.nlm.nih.gov/dailymed/about.cfm) except specifically indicated. Values for affinity (*pK*_*i*_) at D1-5, 5-HT, and adrenergic receptors are from [93, 94] (see also last footnote). The value of D2 indicates D2S and D2L, respectively.

^#^Pharmacokinetic data of lisuride are from [95].

^†^Pharmacokinetic data are followed by transdermal administration.

^§^Pharmacokinetic data are followed by subcutaneous administration.

^‡^ *pK*_*i*_ values of rotigotine are calculated based on *K_i_* values (M) that are described in [93] (*pK*_*i*_ = − log _10_ *K*_*i*_). The value of D4 indicates D4.2, D4.4, and D4.7, respectively.
